# Design and Fabrication of CMOS Microstructures to Locally Synthesize Carbon Nanotubes for Gas Sensing

**DOI:** 10.3390/s19194340

**Published:** 2019-10-08

**Authors:** Avisek Roy, Mehdi Azadmehr, Bao Q. Ta, Philipp Häfliger, Knut E. Aasmundtveit

**Affiliations:** 1 Department of Microsystems, University of South-Eastern Norway, 3184 Borre, Norway; Mehdi.Azadmehr@usn.no (M.A.); Bao.Ta@usn.no (B.Q.T.); Knut.Aasmundtveit@usn.no (K.E.A.); 2 Department of Informatics, University of Oslo, 0373 Oslo, Norway; hafliger@ifi.uio.no

**Keywords:** carbon nanotube, local CNT synthesis, CMOS–CNT integration, microstructures, microheater, CMOS-compatible temperature

## Abstract

Carbon nanotubes (CNTs) can be grown locally on custom-designed CMOS microstructures to use them as a sensing material for manufacturing low-cost gas sensors, where CMOS readout circuits are directly integrated. Such a local CNT synthesis process using thermal chemical vapor deposition (CVD) requires temperatures near 900 °C, which is destructive for CMOS circuits. Therefore, it is necessary to ensure a high thermal gradient around the CNT growth structures to maintain CMOS-compatible temperature (below 300 °C) on the bulk part of the chip, where readout circuits are placed. This paper presents several promising designs of CNT growth microstructures and their thermomechanical analyses (by ANSYS Multiphysics software) to check the feasibility of local CNT synthesis in CMOS. Standard CMOS processes have several conductive interconnecting metal and polysilicon layers, both being suitable to serve as microheaters for local resistive heating to achieve the CNT growth temperature. Most of these microheaters need to be partially or fully suspended to produce the required thermal isolation for CMOS compatibility. Necessary CMOS post-processing steps to realize CNT growth structures are discussed. Layout designs of the microstructures, along with some of the microstructures fabricated in a standard AMS 350 nm CMOS process, are also presented in this paper.

## 1. Introduction

Nanomaterials are a popular research topic as sensing materials for various sensing applications [1,2,3], including gas detection [4,5,6,7,8], due to their compact size [9], high sensitivity [10], low operating temperatures [11], low power consumption [12], etc. Among different nanomaterials, carbon nanotubes (CNTs) offer a very high surface-to-volume ratio [13], as well as exceptional material properties, including mechanical strength [14], metallic or semiconducting electrical conductivity [15], and high thermal conductivity [16] that facilitates CNTs in diverse applications [17,18,19,20,21]. Electrical properties of CNTs are easily influenced during interaction with different gas molecules [10], making these carbon-based nanomaterials an excellent candidate for ultra-sensitive gas sensing. Moreover, functional materials such as Pd and Pt can be deposited on CNTs [22] to enhance their sensitivity and selectivity to various gases, such as NO_2_, NH_3_, CO and O_2_ [23,24]. A CNT-based gas sensor can have small form factor, while it can sense gases even at low concentration exposure at room temperature. Such a sensor is beneficial to numerous applications, including in the food-packaging industry, where the freshness of the food can be monitored by sensing released gases due to food degradation.

**Figure 1:**
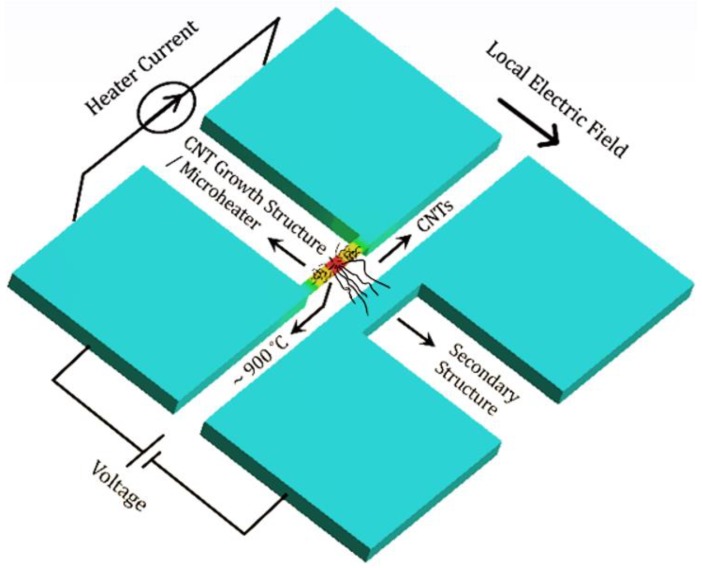
Concept illustration of local carbon nanotubes (CNT) synthesis on microstructures.

To utilize the functionalities of CNTs in smart gas-sensing applications, high-quality, low-noise CMOS front-end circuits are required for signal conversion and processing. A compact gas sensor containing CNTs as sensing material and CMOS readout circuits needs a standard process for direct CNT–CMOS integration. CNTs were already directly synthesized on MEMS structures, as demonstrated by Englander et al. [25], back in 2003. A similar approach can be used to grow CNTs on CMOS structures [26]. Figure 1 illustrates the process of local CNT synthesis on microstructures. The CNT growth structure (microheater) is locally heated by joule heating to generate temperatures in the region of 900 °C. A pre-deposited thin catalyst layer (iron in our process) on the microheater breaks into nanoparticles at such high temperatures, while a carbon-containing precursor gas enters the chemical vapor deposition (CVD) chamber. CNTs start to grow on the microheater when the precursor gas interacts with the catalyst nanoparticles. An electric field set between the CNT growth structure and another neighboring microstructure guides the growing CNTs toward that nearby secondary microstructure. A circuit containing CNTs as variable resistor is formed once the CNTs land on the secondary microstructure. Such a system can potentially work as a gas sensor, since the change in CNT electrical properties can be monitored upon exposure to certain gases.

CNT–MEMS integration acts as a model system for CNT–CMOS integration. CMOS and MEMS technologies have clear similarities, but demonstration of direct CNT integration in CMOS involves a more complex process than in MEMS. Alternatively, CMOS chips can be assembled with CNT–MEMS to demonstrate the ultimate goal of a single chip with sensing and signal-processing capability. However, MEMS–CMOS assembly adds cost and processing steps, which are not compatible with low-cost mass production.

The main challenge for directly growing CNTs in CMOS chips is the high contrast in temperature, since CNT synthesis using our localized CVD process needs around 900 °C, whereas CMOS circuits are at risk to be degraded at temperatures above 300–400 °C [27,28,29]. Therefore, it is important to grow the CNTs locally and keep the CNT growth microstructures thermally isolated. In MEMS, CNTs are directly synthesized on a suspended microstructure by local resistive heating during the CVD process [25,30,31]. However, industrial CMOS fabrication platforms do not allow fabricating suspended structures. For full or partial suspension of any CMOS microstructures, post-processing steps need to be introduced on the CMOS chips.

CNTs can also be synthesized separately at a high temperature and then transferred to the microsystem for integration. Since the CNTs are grown separately, the high temperature requirement for the growth along with other post-growth processes, such as purification or chemical modification, does not have any restrictions in this technique. Among post-growth CNT integration schemes, liquid deposition methods, including dielectrophoresis (DEP) [32] and dip coating [33], are well-known. In general, liquid suspension techniques lack good bonding between the substrate and the nanostructures, and there are possibilities of contamination and sacrificing electrical properties during the protracted process steps [34]. They also often suffer from issues related to non-uniform CNT assembly and misalignment [32,35]. Therefore, it is challenging to realize a reliable and repeatable technique of transferring and assembling CNTs to the microsystem.

In a recent work, Hills et al. [36] proposed a manufacturing methodology for CNTs which combines processing and circuit design techniques to overcome the major challenges related to liquid deposition methods for CNT integration in CMOS. However, the complex nature of this work on CNFET-based digital circuits requires defining a dedicated CMOS fabrication process involving materials such as Pt, Ti for metal layers and SiO_x_, HfO_x_ for PMOS and NMOS passivation layers [36], which are not common is standard CMOS processes. In the case of the DEP process, it provides better alignment and uniformity, but usually has low throughput and requires additional complex metallization steps to achieve better electrical contact between the nanotubes and the electrodes [37]. Seichepine et al. [32] developed CNT integration in CMOS by DEP method with high yield, where 80% of the electrode pairs in the 1024 array of devices only have 1–5 CNT connections per electrode pair; however, these CMOS chips still required extensive metallization post-processing [38]. Local CNT synthesis ensures CNTs with excellent structural quality, but the CMOS-compatible temperature is the main restriction in this integration process [32], and that is what we aim to solve in our approach of CMOS–CNT integration.

The CNT growth structures in CMOS can be fabricated using the polysilicon and metal interconnecting layers. Aluminum (Al) and copper (Cu) are commonly used for metallization in standard CMOS technology [39,40]. These metals, however, are not suitable to serve as microheaters (CNT growth structures) in their original form due to low electrical resistivity and high thermal conductivity. Moreover, aluminum melts at around 660 °C, which is far less than the required 900 °C CNT synthesis temperature. Therefore, we have investigated different binary alloys containing Al or Cu that have material properties suitable for our application. Nickel (Ni) turned out to be the most suitable alloying material for both Al and Cu.

We designed several microstructures suitable for CNT synthesis, using the compatible materials (polysilicon, Al–Ni, and Cu–Ni alloys) in CMOS and performed thermomechanical analyses with the Multiphysics simulation software, ANSYS. Most of these designs involve partially suspended microstructures to balance mechanical stability and high thermal isolation. A few designs involve fully suspended microstructures or no suspension at all. The simulation results presented in this paper show the feasibility of reaching CNT synthesis temperature by local resistive heating, while keeping safe temperatures on the chip for the CMOS circuits. As a result, we also designed a CMOS chip containing different CMOS microstructures with the purpose of local CNT synthesis, and the chips were fabricated using a standard AMS 350 nm CMOS process. Layout designs of some of these CMOS microstructures, as well as optical micrographs of these fabricated structures, are presented in this paper.

## 2. Design Approach for CNT Growth Structures in CMOS

To grow CNTs in CMOS chips by thermal CVD process, a microheater as CNT growth structure is required that can locally generate a hotspot of ~800–900 °C. Therefore, we have investigated the materials commonly used in standard CMOS processes to design efficient microheaters. It is essential to keep the CMOS circuits at a safe temperature, below 300 °C, when the microheater produces the CNT synthesis temperature; hence, the design approach to maintain the CMOS-compatible surrounding temperature is also discussed.

### 2.1. Materials for CMOS Microheaters

The local CNT synthesis temperature was generated by joule heating in the simulations, where an electrical current is passed through a conductor to produce the heat. In our study, we were only considering conduction as a medium of heat transfer, neglecting convection and radiation due their minimal effect in micro scale compared to conduction. The nature of convection can be realized by Rayleigh number (Ra), where higher value of Ra indicates more influence of convective heat transfer, and it was shown that Ra decreases significantly for microscopic structures compared to macroscopic structures; thereby, the effect of convection becomes negligible at the micro level [41]. It was also derived that the heat loss on microstructures due to thermal radiation is less than 1%, even for temperature as high as 1000 °C [41]. Therefore, the dissipated electrical power in resistive heating can be converted to heat by Fourier’s law of heat conduction. We have previously derived an equation for a rectangular heater [42], where the maximum temperature (*T*) at the center can be expressed as Equation (1):

(1) $$T=T_{0}+\frac{I^{2}l^{2}\rho }{8w^{2}t^{2}k}$$

where *T*_0_ is ambient temperature, *I* is the current through the microheater, *ρ* is electrical resistivity, k is thermal conductivity, and *l*, *w*, and *t* are length, width, and thickness of the microheater, respectively.

Materials for microheaters to grow CNTs should have high electrical resistivity (*ρ*) for efficient heating and low thermal conductivity (*k*) to reduce heat loss in the surroundings. In terms of material parameters, the ratio *ρ*/*k* is an important factor in achieving high temperature on the microheaters by resistive heating. This ratio is proportional to the maximum temperature on the microheater, as shown in Equation (1); hence, a higher value of this ratio is desirable.

Standard CMOS technologies offer several metal interconnect layers comprising Al or Cu, along with conductive polysilicon layers. The melting point of Al is much lower than CNT synthesis temperature, and *ρ*/*k* ratio for both Al and Cu are very low for effective resistive heating. Therefore, we investigated several binary alloy options for both Al and Cu. Alloys provide higher electrical resistivity and lower thermal conductivity than the native form of the involved metals, so higher *ρ*/*k* ratio can be obtained. Polysilicon has a very high melting point, as well as high *ρ*/*k* ratio, making it the most suitable material in CMOS for our microheater application. AMS 350 nm CMOS process uses two polysilicon layers, one polysilicon layer (poly-1) being thicker than the other (poly-2) [43]. A thinner polysilicon layer (poly-2) results in higher electrical resistivity and can also be mechanically beneficial for suspended microstructures, due to less residual stress compared to thicker structures [44].

In a previous study, we showed Cu–Ni alloy to be the best-suited microheater material among other Cu alloys [42]. It should be noted from Equation (1), microheater thickness square (*t*^2^) is inversely proportion to microheater temperature, and thickness of the microheater depends on the alloy composition. Although a 40% Cu–60% Ni composition (at.%) has higher *ρ*/*k* ratio, the lower microheater thickness of 70% Cu–30% Ni alloy makes it a more efficient material in this application. A comparison of the relevant material properties (at CNT growth temperature) of copper and its suitable alloys are presented in Table 1.

In this study, we investigated suitable aluminum alloys for designing metal microheaters since we have designed and fabricated our CMOS chip in AMS 350 nm CMOS process, which offers aluminum metal layers. It should be noted that Al melts at a far lower temperature (~660 °C) than the required CNT growth temperature (~900 °C). Several compositions of Al–Ni alloys, however, have a melting point higher than 900 °C. Based on the material properties, tri-nickel aluminide (Ni_3_Al) is the most suitable aluminum alloy for realizing metal microheaters in the AMS 350 nm CMOS process. A comparison of the relevant material properties (at CNT growth temperature) of aluminum and its suitable alloys are presented in Table 2.

Ni_3_Al alloy has excellent mechanical strength and wear-resistance at high temperatures [49]. The features of this alloy are well-suited for high-temperature applications, such as promising applications in aircraft engines as structural bulk material [50] and MEMS applications (such as micro-actuators) as thin foils [51,52]. This intermetallic phase also has a potential application in microchemical systems as catalysts (microreactors) [52,53].

**Figure 2:**
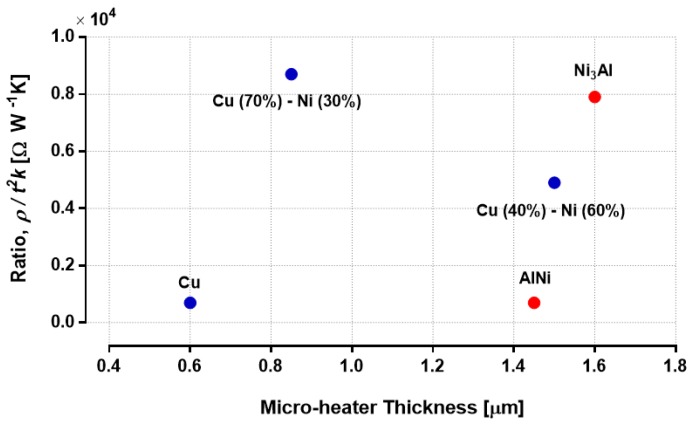
Influential ratio (*ρ*/*t*^2^*k*) for maximum microheater temperature plotted against total microheater thickness associated with the material.

In Equation (1), length (*l*) and width (*w*) of the microheater are defined by our design; current (*I*) is a variable during the joule heating process, and the heater thickness (*t)* and material properties (*ρ*, *k*) are CMOS-process-dependent parameters. The ratio *ρ*/*t*^2^*k* from the equation is directly proportional to the maximum microheater temperature. Although the value of this ratio depends on the CMOS process, it is modified for the metal microheaters when they are transformed into alloys. Therefore, we plotted this ratio in Figure 2 for five different potential materials from Table 1 and Table 2. The values of *ρ* and *k* are taken at CNT synthesis temperature. Thickness of the alloys are calculated based on a 0.6 μm thick Al or Cu layer, where atomic weights and mass densities of the associated materials are taken into account. A higher value of the ratio *ρ*/*t*^2^*k* corresponds to a more efficient microheater, allowing room for reducing the *l*/*w* ratio to achieve mechanically more robust designs for suspended heaters. Based on the plot in Figure 2, Cu (70%)–Ni (30%) and Ni_3_Al alloys are the most suitable metallic microheater materials among the available options in CMOS for the CNT synthesis application.

The choice between metal and polysilicon microheaters should be based on suitability in CMOS post-processing, although polysilicon has significantly better material properties in terms of using them as microheaters for local CNT synthesis. The metal options are CMOS-process-dependent. Cu–Ni (70%–30%) is the preferred alloy composition if CMOS metal layers are made of Cu, whereas Ni_3_Al alloy is suitable as the microheater material in case Al is used in the metallization layer for the CMOS process. Among the polysilicon options, a thinner polysilicon layer with higher electrical resistivity is more efficient as a microheater.

### 2.2. CMOS-Compatible Chip-Surface Temperature

High temperature (~800–900 °C) is necessary to grow CNTs of small diameter (below 10 nm), which ensures aligning of those CNTs by local electric field [54,55] so that they can be suspended between the two microstructures. Therefore, the microstructure/microheater for synthesizing CNTs needs to produce a local hot region of 900 °C. Even if the synthesis temperature is generated by local resistive heating, the entire chip-surface temperature rises higher than the CMOS-compatible temperature due to thermal conduction, since the microstructures in CMOS are surrounded by dielectric layer. Hence, the target is to achieve high enough thermal gradient around the microheater so that CMOS transistors can reside at safe temperatures.

Maximum thermal gradient around the microheaters can be reached by completely etching the dielectric layer underneath those growth structures; however, the microheaters would suffer from undesirable mechanical deformation. Therefore, it is beneficial to keep some dielectric layer underneath the microheaters as a mechanical support. Having dielectric support underneath also means a heat conduction path to the bulk of the chip. It is trade-off between achieving a high thermal gradient and the keeping mechanical integrity of the microheater. So, our goal is to under-etch a sufficient amount of dielectric to get the required thermal isolation for maintaining CMOS-compatible temperatures, while avoiding destructive microheater deformation.

It is important to note that the high microheater temperature is only required for growing CNTs. Therefore, the concern of compatible temperatures for CMOS transistors is limited to the duration of the one-time CNT synthesis process, which takes around 5 min. The CNT-based sensor can operate at room temperature during gas-sensing operation.

## 3. Thermal Analysis of CMOS Microstructures

We designed several microheaters using the available materials in the standard CMOS process and performed thermoelectric and thermomechanical simulations using Multiphysics simulation software, ANSYS. All microheaters are mechanically stable according to the thermomechanical analysis; however, in practice, the stability of suspended microheaters is significantly affected by the wet etching process. Most of the designed microheaters are partially suspended, meaning a limited amount of the dielectric layer stays underneath the heaters. The simulated models also have a bulk silicon layer and a chip-packaging material layer below the dielectric layer. Room temperature is considered in the bottom surface of the package material as a boundary condition, which is a practical assumption if a heat sink is used during the process. The silicon substrate will be in direct contact with a heat sink if the CNT synthesis process is carried out at the wafer-level during a potential high-volume production, which will further facilitate in maintaining CMOS-compatible temperature.

We have previously worked on thermomechanical simulations of Cu–Ni and polysilicon microheaters [42,56]. However, in the later stage, we chose a standard AMS 350 nm CMOS technology to design and fabricate the microstructures (detailed in Section 4), which uses aluminum as the interconnecting metal layers. Therefore, in this article, we investigated Al-based alloys suitable for our application, which is reported in Section 2.1, and, consequently, the simulations of the Ni_3_Al alloy microheaters are presented in this section. During the CMOS chip design stage, we also found that our previous partially suspended microheater (PSM)-design approaches [42,56] would have some challenges in post-processing and CNT growth, which are discussed in Section 5.3. Hence, we used new PSM-design approaches to overcome those challenges and performed thermal simulations on polysilicon and Cu–Ni microheaters, accordingly. These new simulation results of the mentioned microheaters are also presented in this section.

### 3.1. Polysilicon Microheaters

The polysilicon microheaters are designed with two different layers: poly-1 and poly-2. In the simulations, the considered sheet resistances of poly-1 and poly-2 are ~6 Ω/sq and ~18 Ω/sq, respectively The fabricated polysilicon microheaters presented in Section 4.2 have ~10 Ω/sq and ~50 Ω/sq sheet resistances for poly-1 and poly-2, respectively. The conservative sheet-resistance values in the simulation mean the fabricated CMOS microstructures should provide better performance as heaters compared to the simulation results. All polysilicon heaters are simulated with 0.85 μm thickness, even though their actual thickness in a CMOS process will be 3–4 times lower. Therefore, the polysilicon heaters will have higher resistance in practice, which is beneficial for resistive heating. In the FEM simulations, higher polysilicon thickness helps to reduce the number of elements for meshing and, consequently, limits the processing time of each simulation.

#### 3.1.1. Polysilicon Microheaters with Limited Under-Etching

**Figure 3:**
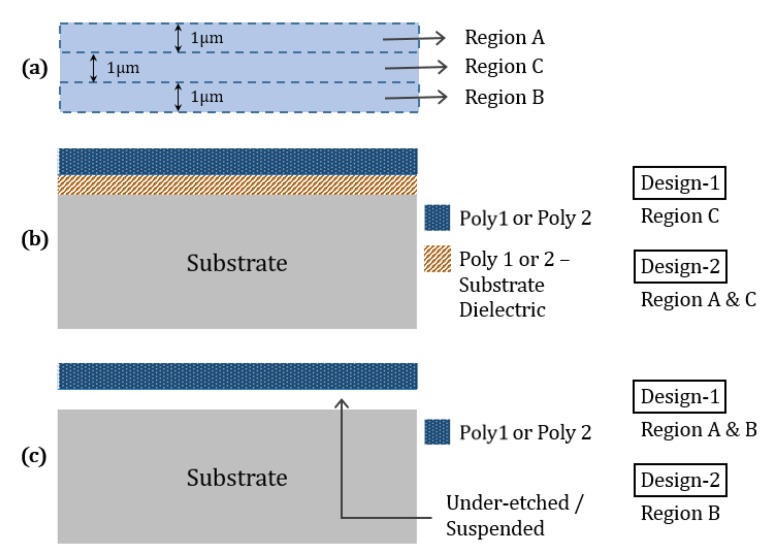
Partially suspended polysilicon microheater design illustrations. (**a**) Microheater surface area; (**b**) cross-sectional view of non-suspended region, and (**c**) cross-sectional view of suspended region.

The microheaters can be partially suspended along their entire length; Figure 3 reveals the suspended and non-suspended areas. Figure 3a illustrates the microheater surface area, which was divided into three regions. We have presented two PSM designs in this section, where region C is non-suspended and region B is suspended in both designs. A cross-sectional illustration of non-suspended and suspended microheater areas is shown in Figure 3b,c, respectively. The non-suspended regions have a dielectric layer underneath that mechanically supports the microheater.

The designed polysilicon PSMs are 30 μm long and 3 μm wide, giving around 60 and 180 Ω resistance to poly-1 and poly-2 heaters, respectively, when they reach the CNT synthesis temperature. In these models, both region A and B are suspended, keeping dielectric support in region C for design-1, while only region A is suspended for design-2. In design-2, region B can be blocked from under-etching by putting a mask using the top metal layers of the CMOS design on one side of the microheater, as detailed in Section 5.3.

**Figure 4:**
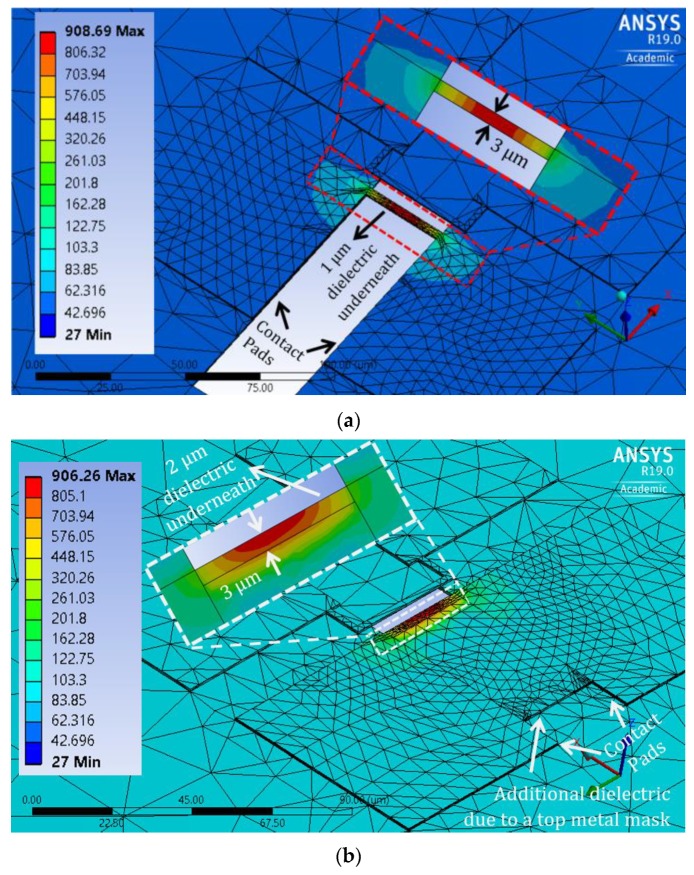
Thermal–electric simulation results of poly-1 partially suspended microheater (PSM) designs: (**a**) design-1 and (**b**) design-2.

Figure 4 shows the thermal simulation results for both designs, where poly-1 was used as the heater material. The microheaters locally generate the required CNT synthesis temperature in their centers. Design-1 (Figure 4a) achieves a high thermal gradient around the heater due to the 1 μm dielectric etching on both sides (region A and B) of the 3 μm wide heater, leaving 1 μm wide dielectric in the middle (region C). Design-2 (Figure 4b) has a 1 μm wide suspension on one side of the heater (region B), leaving a 2 μm wide dielectric layer (region A and C) underneath the heater. Suspended microheater regions produce a higher thermal gradient around them. Therefore, the microheater in design-2 has lower thermal isolation than the microheater in design-1. Moreover, since a top metal layer blocks heater under-etching on one side (region A), the dielectric layer will remain in the blocked region near that side of the heater, as indicated in Figure 4b, creating more heat-conduction paths. Even with lower thermal isolation of the heater, the average surrounding surface temperature in design-2 is near 100 °C. Therefore, both poly-1 PSM designs maintain the CMOS-compatible temperature in the chip, while producing a local hotspot for CNT synthesis. From a mechanical viewpoint, design-2 is more preferable over design-1, as it involves less dielectric etching underneath the heater. In terms of power consumption, design-1 (~35 mW) is more efficient than design-2 (~80 mW).

**Figure 5:**
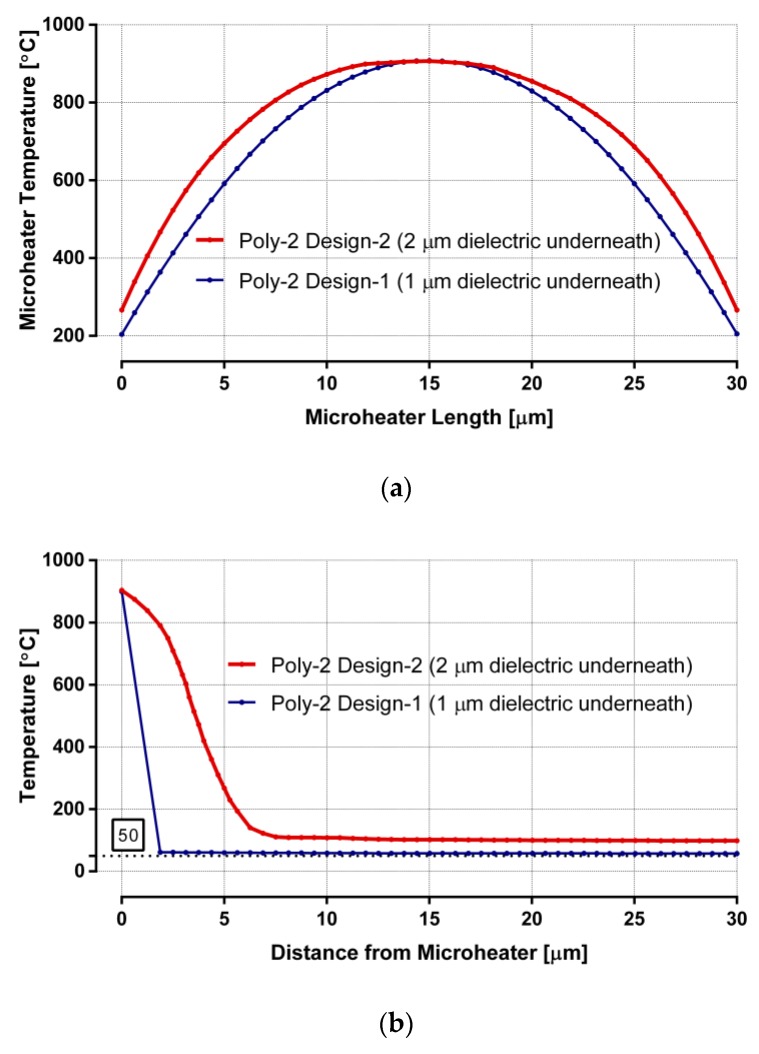
Thermal analysis of partially suspended poly-2 designs: (**a**) microheater temperature along its length; (**b**) temperature distribution from the center of the heater to the surrounding surface.

The plots in Figure 5 show the thermal-simulation results of poly-2 microheaters for both design-1 and design-2. Temperatures along the length of the heaters are plotted in Figure 5a. The graphs form parabolic shapes (as predicted by Equation (1)) with the maximum temperature (~900 °C) at the center of the heaters, while CMOS-compatible temperatures are reached even at both ends of the heaters. The power requirements for these heaters are similar to the corresponding poly-1 designs. Plots in Figure 5b indicate the temperature drops when moving away from the heaters. High thermal gradient around the heaters is apparent in the plots. Surface temperature in design-1 reaches ~50 °C, within 25 μm away from the heater, achieving an average thermal gradient of 34 °C per micrometer around the heater. In design-2, the surface temperature becomes ~100 °C, within 25 μm away from the heater, indicating an average thermal gradient of 32 °C per micrometer around the heater.

#### 3.1.2. Non-Suspended and Fully Suspended Polysilicon Microheaters

Two polysilicon microheaters were modelled with a reduced length of 6 μm for simulating non-suspended and fully suspended designs. The widths of the non-suspended and fully suspended designs are 1 and 2 μm, respectively. In the case of the non-suspended design, reduced surface area of the microheater means less of a heat-conduction path through the dielectric layer. The reduced length for a fully suspended microheater offers lower risk of deformation. Although it may still suffer from buckling and stiction, the width of this design was extended to 2 μm for improving mechanical steadiness. At around 900 °C, resistances of fully suspended and non-suspended designs are ~55 and ~110 Ω, respectively.

**Figure 6:**
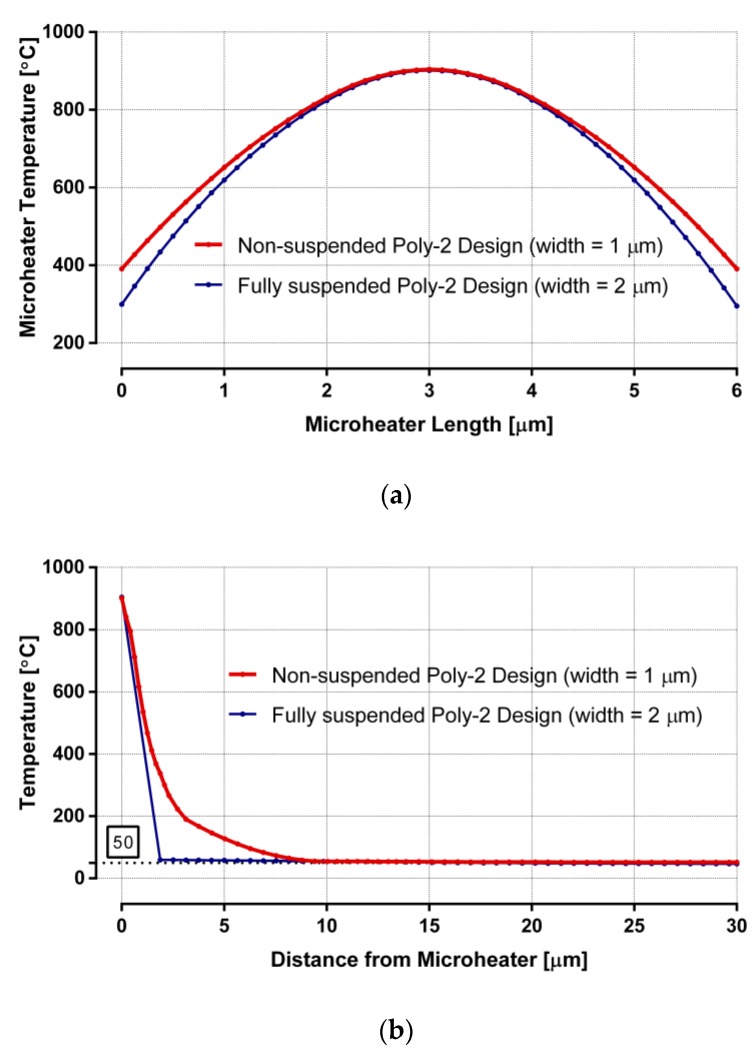
Thermal analysis of non-suspended and fully suspended poly-2 designs: (**a**) microheater temperature along its length; (**b**) temperature distribution from the center of the heater to the surrounding surface.

These microheaters are only designed with poly-2 layer since it has higher electrical resistivity among the polysilicon options. Thermal responses of both non-suspended and fully suspended designs are plotted in Figure 6. Plots in Figure 6a present the temperatures of the heaters, both having a 2 μm long region in the center, where the heaters reach more than 800 °C, an optimum region to grow low-diameter CNTs. Plots in Figure 6b show that the average surrounding chip temperature reaches far below CMOS-compatible temperature for both designs. Both designs have a very high thermal gradient around the heaters, indicated by the sharp temperature decrease. They also have a similar power requirement of ~20 mW. The non-suspended heater is a clear favorite among these designs, as it avoids mechanical deformation and does not require any dielectric under-etching steps during the CMOS post-processing.

### 3.2. Tri-Nickel Aluminide (Ni_3_Al) Microheaters

**Figure 7:**
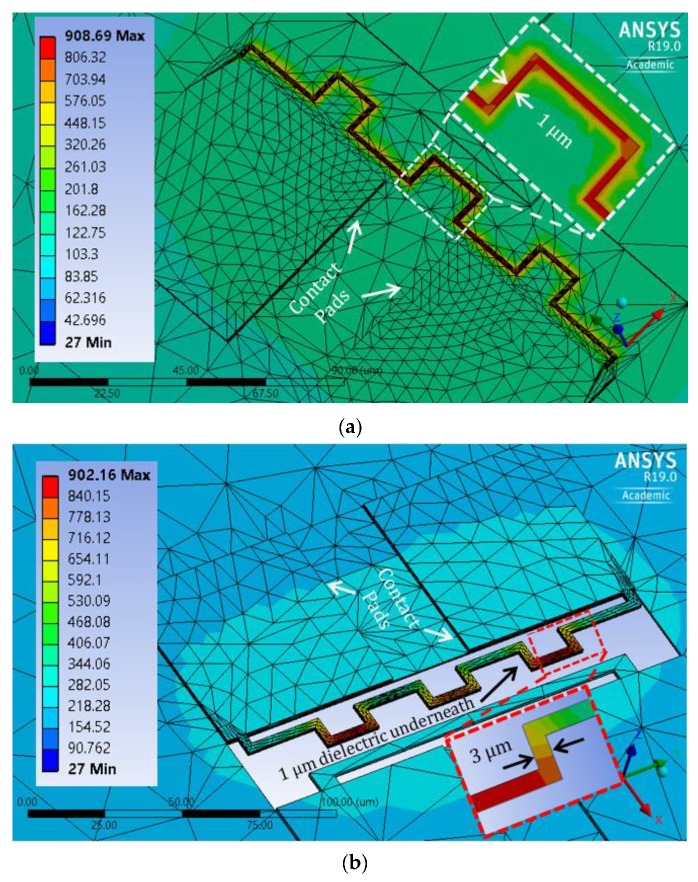
Thermal–electric simulation results of Ni_3_Al designs: (**a**) non-suspended and (**b**) partially suspended.

For the standard CMOS process with Al metallization, microheaters were designed and simulated using tri-nickel aluminide (Ni_3_Al). Figure 7 shows the thermal simulation results for both non-suspended and partially suspended designs. Around a 1 μm thick layer of Ni needs to be deposited on a 0.6 μm thick Al layer to obtain the Ni_3_Al phase, considering the atomic weights and mass densities of Ni, Al, and Ni_3_Al. Therefore, these microheaters are designed with a 1.6 μm thickness. The width of non-suspended design in Figure 7a is 1 μm, whereas PSM (with limited under-etching along the length, similar to design-1 from previous section) in Figure 7b has a 3 μm width. PSMs with a greater width would yield higher mechanical stability in the under-etched design, while lower width of the non-suspended design results in less heat conduction on the substrate.

For efficient resistive heating, the microheaters need sufficiently high resistance. Hence, the length of the metal heater designs was extended to ~240 μm for increasing heater resistance. The PSM design had ~15 Ω room temperature resistance, which goes above 35 Ω at the CNT synthesis temperature due to increase in Ni_3_Al resistivity at the elevated temperature. The non-suspended design has a 3-times-higher heater resistance due to a 3-times-lower width. The non-suspended design (Figure 7a) produces a chip surface temperature above 300 °C, due to lack of thermal isolation of the heater. This post-processing temperature is not suitable for CMOS circuits. However, the partially suspended design (Figure 7b) has sufficient thermal isolation for the heater, resulting in a CMOS-compatible surrounding temperature of ~200 °C. It requires around 350 and 150 mW power for the non-suspended and partially suspended microheaters, respectively, to generate the CNT synthesis temperature.

### 3.3. Cupronickel (Cu–Ni) Microheaters

Cu–Ni microheaters were designed and simulated for the CMOS processes that use Cu as interconnecting metal. Material properties of 70% Cu–30% Ni alloy composition is used for the heaters. Considering the atomic weights and mass densities of Cu and Ni, the heaters are modelled with a 0.85 μm thickness. Depositing 0.25 μm thick Ni on top of a 0.6 μm thick Cu layer is suitable for achieving 70% Cu–30% Ni binary alloy composition upon annealing. The length and width of the heaters in both non-suspended and partially suspended designs are kept same as the Ni_3_Al microheater designs. Heater resistances of Cu–Ni designs are also similar to the Ni_3_Al heaters, as sheet resistances of both Cu–Ni and Ni_3_Al heaters are alike at near CNT synthesis temperature.

**Figure 8:**
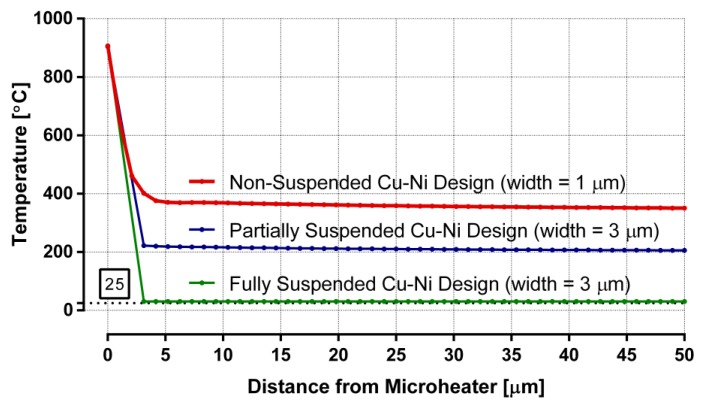
Temperature distribution from the center of different cupronickel microheaters to the surrounding surface.

Microheater temperatures, along with the surrounding surface temperatures, are plotted for non-suspended, partially suspended, and fully suspended Cu-Ni designs in Figure 8. All these heaters have a local hot region of ~900 °C for growing CNTs, and the temperature starts to drop when moving away from the heaters. The non-suspended design reaches a CMOS-incompatible surface temperature of ~350 °C, while the PSM design manages to achieve a CMOS-compatible chip surface temperature of ~200 °C. Average surrounding temperature of the fully suspended Cu–Ni design drops down to near room temperature. At 25 μm away from the heaters, the average thermal gradients around the non-suspended, partially suspended, and fully suspended Cu–Ni microheaters are 22, 28, and 35 °C per micrometer, respectively. The power requirements of non-suspended and partially suspended designs are similar to the respective Ni_3_Al designs, while the fully suspended heater consumes less than 5 mW of power. Even though the fully suspended heater has the maximum thermal isolation and lowest power consumption, the design is not practically feasible, as it is prone to mechanical deformation. Therefore, PSM is the most suitable among the Cu–Ni designs for our application.

## 4. Fabricated CMOS Microstructures for CNT Synthesis

We selected a standard AMS 350 nm CMOS process to fabricate microstructures for locally synthesizing CNTs. In this CMOS technology, aluminum is used for metallization, and copper is not available. Therefore, aluminum and polysilicon layers were used for designing and fabricating the CNT growth structures. The designed 3 mm × 3 mm CMOS chip contains a variety of microstructures, some of which were presented in one of our previous papers [57]. In this article, we present the layout and optical micrograph of the designs relevant to the simulated designs we have presented in Section 3.

### 4.1. CNT Growth Structures Using Aluminum Layers

**Figure 9:**
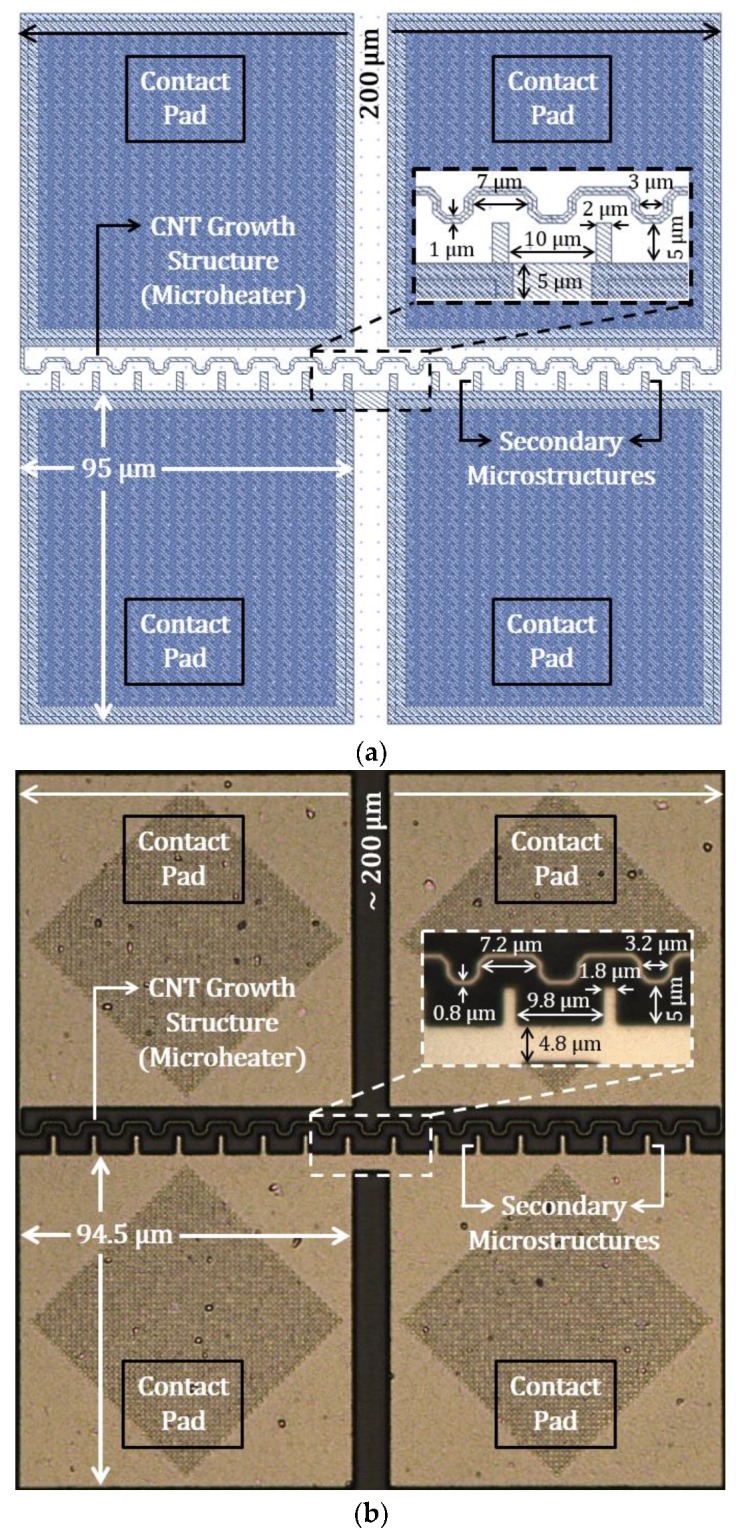
Aluminum CNT growth structures in AMS 350 nm process: (**a**) layout design and (**b**) optical micrograph.

Layout design and optical micrograph of the fabricated aluminum microstructures are presented in Figure 9a,b, respectively. The top metal layer was used for the aluminum designs. In this wave-shaped microheater design, the length of the heater is efficiently increased to over 350 µm. The designed width of the heater is 1 µm, resulting in a measured resistance of ~50 Ω. To generate CNT synthesis temperature, Ni needs to be deposited for producing Ni_3_Al binary alloy, which will increase both thickness and electrical resistivity of the microheater. Several rectangular beams are used as secondary microstructures, which go in between the waves of the microheater for producing a high electric field at the beam tips during the CNT growth process, so that some of the synthesized CNTs can be connected to the secondary structures after being guided by the local electric field. The contact pads for the microstructures have a surface area of 95 µm × 95 µm, providing sufficient area for wire bonding. Dimensions measured from the optical micrograph of the fabricated Al microstructures reflect the corresponding dimensions of the layout design.

We attempted thermal microscopy of the CMOS microheaters using a high-performance infrared camera, FLIR A6750sc MWIR with a 4X microscopic lens. While the CMOS-chip-surface temperatures can be measured easily, measuring temperatures on the microheaters was an issue. The camera operates at 3–5 µm wavelengths, with the minimum spot pixel size of 3 µm × 3 µm. The camera also needs an array of 3 × 3 pixels for an adequate thermal measurement. Hence, the narrowest microstructure dimension needs to be at least ~9 µm to obtain acceptable measurements, while most of our microheaters only have ~1 µm widths. Therefore, we did not get accurate temperature data for the microheaters, as the obtained temperature values of the microheaters have an average of the heater temperature and surrounding area temperature of the heater.

**Figure 10:**
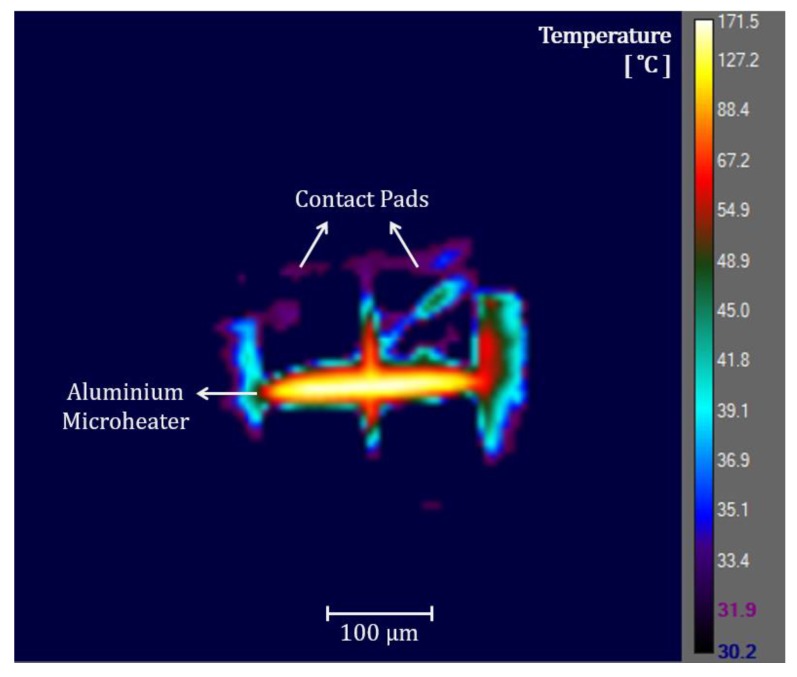
Thermal micrograph of an aluminum microheater on a CMOS chip.

Figure 10 shows an infrared image of the Al microheater presented in Figure 9. The heater temperature was controlled by joule heating. Although maximum temperature on the microheater appears to be ~170 °C in Figure 10, the actual heater temperature was much higher. This was confirmed since the Al microheater was melted at the region of maximum temperature when the electrical current through the heater was slightly increased. Therefore, we could estimate that the actual temperature on that hottest spot of the aluminum microheater was ~660 °C (melting temperature of Al). The thermal measurement of the surface surrounding the microheater was accurate, which shows a temperature of ~30 °C. Although the temperature measurement on the microheater was not accurate due to resolution limitation, it is still a positive indication that the surrounding CMOS chip area remains at near room temperature while the Al heater reaches near melting temperature. This shows a convincing sign toward the agreement with the thermal simulation results and maintaining CMOS-compatible temperatures on the chip surface when the microheaters would reach CNT growth temperatures.

### 4.2. CNT Growth Structures Using Polysilicon Layers

**Figure 11:**
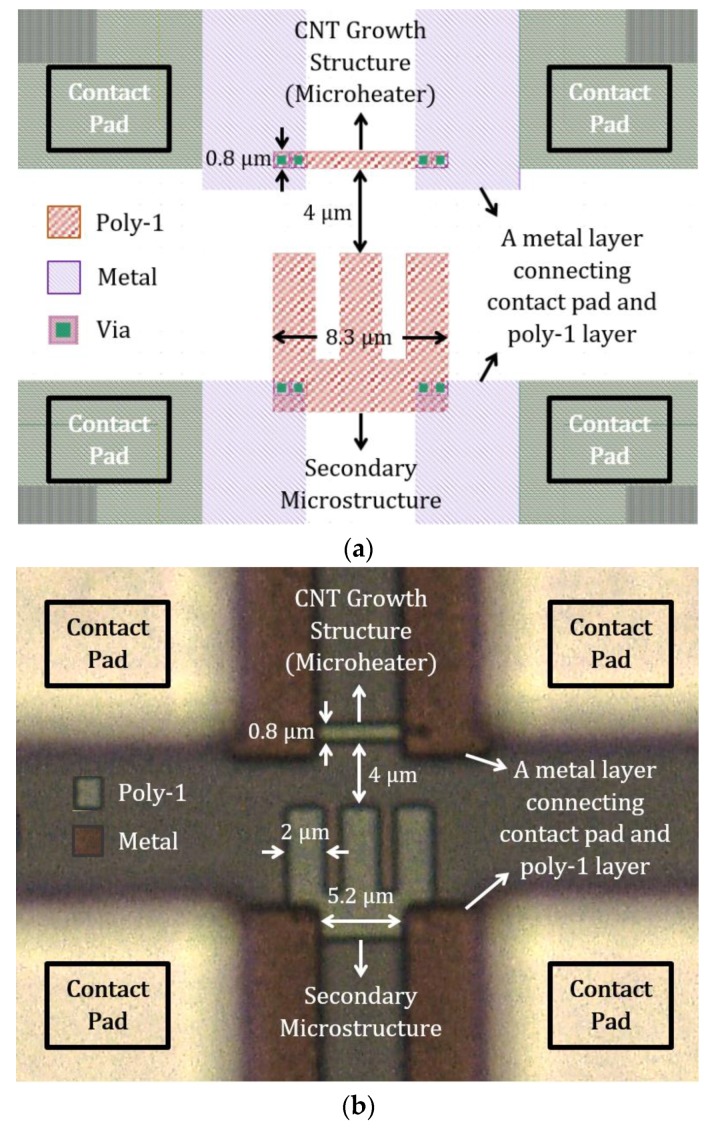
Poly-1 CNT growth structures in AMS 350 nm process: (**a**) layout design and (**b**) optical micrograph.

A poly-1 design for local CNT synthesis application is shown in Figure 11. The CMOS layout design in Figure 11a illustrates all the comprised layers where the vias provide access to metal layers for the polysilicon structures. The extended metal layer aids in designing an 8.3 µm long heater, while keeping a 15 µm distance between the contact pads. The microheater is 0.8 µm wide and has a measured resistance of ~100 Ω. The heater and secondary structures are 4 µm apart. Dimensions of the microstructure layouts are properly translated to the fabricated polysilicon structures, as seen in the optical micrograph in Figure 11b. This polysilicon microheater has comparable dimensions to one of the simulated heaters in Section 3.1.2.

**Figure 12:**
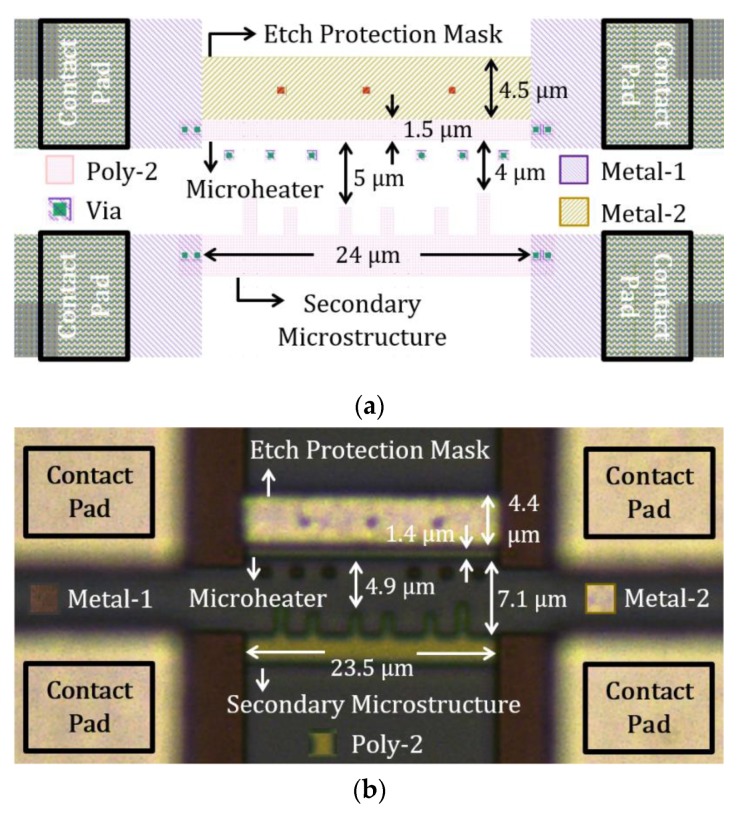
Poly-2 CNT growth structures in AMS 350 nm process: (**a**) layout design and (**b**) optical micrograph.

Figure 12a shows the layout of a poly-2 design, and Figure 12b shows the corresponding optical micrograph of the fabricated CMOS microstructures. The design involves a poly-2 layer, two different metal layers, and vias for electrical connection between layers. Some additional vias are purposely placed in front of the microheater to limit dielectric etching in those regions. In this design, a top metal layer (metal-2) was used as an etch protection mask; hence, the dielectric layer underneath that metal layer is not etched. This poly-2 design resembles a simulated model (design-2) from Section 3.1.1, where the heater is only under-etched from the side facing secondary microstructures. The heater is ~25 µm long and 1.5 µm wide. It has a high measured resistance of ~1 KΩ due to high poly-2 resistivity. The optical micrograph shows the fabricated microstructures, which were rendered well from the designed CMOS layout.

## 5. Discussion

Our fabricated CMOS chips need to go through several post-processing steps for growing CNTs. The process complexity will vary depending on the microheater design; non-suspended designs are easier to realize than the suspended ones. For the metal microheaters, alloying is an added step, where the process complexity also differs among the two preferred alloy options. Under-etching approaches of different partially suspended microheaters are included in this part. In prior publications from our group, synthesized CNTs on custom-designed PolyMUMPS [30,58] and SOIMUMPS [31,54] microstructures were presented with relevant SEM and S(T)EM analyses. Polysilicon CNT growth structures used in the PolyMUMPS process have good resemblance with CMOS for CNT synthesis process demonstration.

### 5.1. CMOS Post-Processing to Realize CNT Growth Structures

Necessary CMOS post-processing to synthesize CNTs on a CMOS chip can be divided into two main stages. The initial stage involves etching the dielectric layers for exposing and under-etching the CNT growth structures in CMOS to achieve high thermal isolation around them. In the case of metal microheaters, this stage also includes making relevant binary alloys. The final stage of the post-processing involves depositing a catalyst layer and growing CNTs on the CMOS microstructures by local thermal CVD process.

The metal and polysilicon layers of a standard CMOS chip are covered with a dielectric layer for electrical isolation, and the entire chip surface is covered with passivation layer(s) for protection from surroundings. In CMOS chip design, we can purposely select regions where the passivation layer(s) can be avoided. It enables an exposed top metal layer surrounded by a dielectric layer. Therefore, the CNT growth structures made from the top metal layer will be readily exposed and available for nickel deposition to produce the alloys.

Nickel can be deposited on the metal tracks (Al or Cu) by evaporation, sputtering, electroplating, or electroless plating. Evaporation and sputtering will need dedicated masks for patterning Ni deposition. Electroless plating can be selective toward deposition on exposed metal tracks, whereas electroplating gives selective deposition on exposed metal tracks that are electrically connected to the electrodes in the electroplating baths. Hence, with an appropriate design, mask-less Ni deposition is possible with plating techniques, where electroplating gives the highest design freedom. Alloying of the Al/Ni bi-layer into homogenous Ni_3_Al is performed through local resistive heating, passing a current through the microheater while in inert atmosphere. The method is similar to that used in the formation of a desired composition of cupronickel alloy.

After the alloying process, the metal microheaters are partially suspended for sufficient thermal isolation during resistive heating. Dry etching by RIE (Reactive Ion Etching) can be performed for anisotropic dielectric etching, followed by a wet etching process to partially release the microheaters. The amount of microheater under-etching depends on the wet etching duration for a specific chemical etchant. Therefore, the required amount of heater suspension can be achieved by controlling the etching duration.

In the case of polysilicon heaters, a thick layer of dielectric is removed by RIE to expose the polysilicon layers. Only this step is required in the initial post-processing stage for non-suspended heaters. However, partially or fully suspended microheaters need an additional step of wet dielectric etching. Even though RIE is anisotropic, it can still cause some heater undercuts, which can be a desirable effect for our application. The wet etching step will no longer be required if the microheaters have sufficient undercuts to obtain required thermal isolation.

Once the CNT growth structures are prepared, a thin catalyst layer (~5 nm) is deposited in the beginning of the final post-processing stage. Iron or nickel is commonly used as the catalyst material, which can be deposited by thermal evaporation, e-beam evaporation, or sputtering. The thin metal layer transforms into nanoparticles at ~900 °C, during resistive heating of the CNT growth structure in the CVD chamber. CNTs start to grow when a carbon-containing precursor gas released in the chamber interacts with the catalyst nanoparticles.

The mentioned CMOS post-processing steps are needed at the research level since they will be performed on individual CMOS chips. However, those process steps may be included in a dedicated process designed for CNT-to-CMOS integration, which can be carried out in wafer-level fabrication for manufacturing commercially viable CNT-based sensors. Zhou et al. [26] previously demonstrated CNT growth on polysilicon structures in CMOS involving various post-CMOS microfabrication processes that we avoid in our approach, with the vision to incorporate the CNT synthesis process in a CMOS technology at wafer-level.

### 5.2. Tri-Nickel Aluminide vs. Cupronickel Microheaters

Forming a tri-nickel aluminide alloy may be more challenging than cupronickel alloy, because of the native oxide layer on aluminum, with Al having a lower electrochemical potential than Cu. Moreover, during the formation of a Ni_3_Al alloy, undesirable intermetallic alloys (such as Al_3_Ni, Al_3_Ni_2_) with a melting temperature lower than 900 °C could also form. A detailed process development is needed to ensure a final microheater composition compatible with high-temperature operation. A certain flexibility in the composition is allowed, however, as both the Ni and the AlNi phases have even higher melting temperatures than that of Ni_3_Al. For Cu–Ni, the exact composition is not a critical parameter, since Cu and Ni form a solid solution, being completely miscible.

Electrical resistivity to the thermal conductivity ratio (*ρ*/*k*) of Ni_3_Al is almost twice that of Cu–Ni (70%–30%) at CNT synthesis temperature (~900 °C). A higher *ρ*/*k* ratio is desirable for the efficient resistive heating of microheaters. However, the thickness of a Ni_3_Al microheater is much higher than that of a Cu–Ni (70%–30%) microheater for a specific Al or Cu thickness, which reduces a Ni_3_Al microheater’s resistance and, consequently, makes it less efficient for heat generation by resistive heating. Considering both alloy formation challenges and material efficiency as a microheater, a CMOS process using Cu for metallization is preferred over the one using Al.

### 5.3. Under-Etching Approaches for PSMs

Two different PSM designs were presented in Section 3.1.1. Design-1 will have limited under-etching along the length from both sides of the heater, whereas design-2 will be under-etched from one side since the other side has a metal mask for avoiding dielectric etching. Our fabricated polysilicon heater in Figure 11 is suitable for design-1 under-etching approach, and the heater in Figure 12 matches the under-etching strategy for design-2. For both designs, under-etching can be obtained by isotropic wet etching, and the amount of suspension depends on the wet etching duration. Different etching times should be attempted to find the optimum duration for the wet etching process to get the required amount of under-etching. The wet etching process may not even be required if the heaters have undercuts within a desired range during the dry etching process, as discussed in Section 5.1.

No mask is needed for the etching process of design-1, but design-2 has a built-in etch protection mask. A top metal block (interconnect layer in CMOS) is placed on one side of the polysilicon microheater, which will act as mask to protect that side of the heater from etching. Therefore, the dielectric layer beneath that metal block will remain. As a result, during the resistive heating process, heat from the microheater will also conduct to the surroundings through the dielectric from that side, which would contribute in making the chip from design-2 hotter than that from design-1.

**Figure 13:**
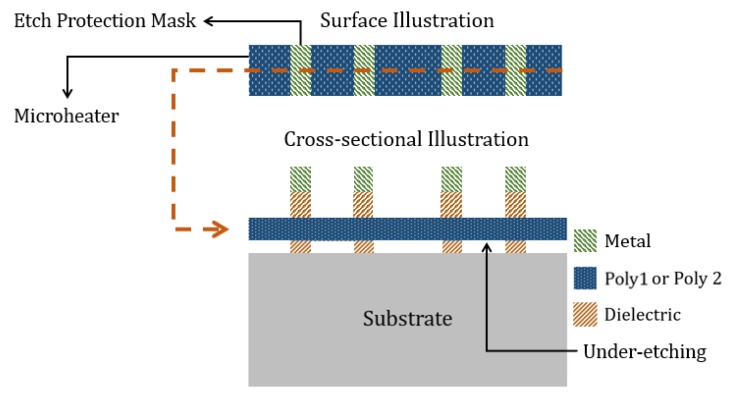
Surface and cross-sectional illustration of a selectively under-etched PSM design.

We have previously designed PSMs using selective patterned under-etching [56]. The surface and cross-sectional view of such a design is shown in Figure 13. Patterned microheater under-etching also needs metal blocks as built-in masks, which will have dielectric material beneath them. Therefore, the top surface of the microheater will be covered with dielectric on the areas where metal blocks are placed above them, as seen in the cross-sectional illustration of Figure 13. This would cause further heat conduction through the top dielectric layer when the microheater is activated, making the chip relatively hotter. Moreover, CNTs cannot grow on the microheater surface, which is covered with dielectric. The improved PSM design approaches presented in this paper solve these issues. Although the microheaters with limited under-etching along their length would be more vulnerable to thermomechanical stress compared to heaters with patterned under-etching, the prior etching approach is still preferred from an overall perspective for PSM designs in our CNT synthesis application.

## 6. Conclusions and Future Work

We presented several designs of CNT growth microstructures (microheaters) using the materials available in standard CMOS process. Polysilicon is the most suitable material in CMOS to serve as a microheater. The metal options (Al or Cu) need to be alloyed with Ni to produce efficient microheaters for the CNT synthesis application. Tri-nickel aluminide and cupronickel (70% Cu–30% Ni) were chosen as the binary alloys from the investigated materials.

Thermal analyses of the CMOS microheaters show that they can have CNT synthesis temperature (~900 °C) on their surface, while having a high thermal gradient to achieve a CMOS-compatible temperature (below 300 °C) on the remaining chip area. Most of the designed microheaters need partial suspension to obtain necessary thermal isolation for CMOS compatibility. The heaters should have sufficient under-etching to obtain a high thermal gradient around them, while avoiding significant mechanical deformation.

CNT growth structures were fabricated in a standard AMS 350 nm CMOS process, using aluminum and polysilicon layers. The microstructures are properly realized in the CMOS chip, as seen from the presented layout designs and optical micrographs of the fabricated structures. The CMOS chips are required to undergo several post-processing steps to prepare the microstructures for local CNT synthesis.

The metal microheaters (Al or Cu) on the fabricated CMOS chips are already exposed; thus, they need alloying as an initial post-processing step, followed by partial dielectric under-etching, using dry and wet etching methods. The polysilicon microheaters need to be exposed first by dry etching of dielectrics and may require wet etching for partial suspension if there are insufficient undercuts on the heaters during the RIE process. The wet etching process is also avoided if the non-suspended polysilicon microheaters manage to obtain required thermal isolation. Once the microheaters are prepared, a thin catalyst layer needs to be deposited on the CMOS chip before carrying out the CVD process for growing CNTs on the microheaters in the final post-processing step.

We already have an established process for locally synthesizing CNTs on MEMS growth structures. However, the CNT growth structures in CMOS need additional processing steps, as discussed for enabling them to obtain the CNT synthesis temperature in a CMOS-compatible environment. These post-processing steps are currently being developed in our cleanroom, using the fabricated CMOS chips. Our next goal is to locally grow CNTs on the dedicated microstructures in the custom-designed CMOS chips. Direct CNT–CMOS integration will be established once the CNTs are suspended between the CMOS microstructures, and the CNT-based prototype sensor can be tested for gas sensing.

## Figures and Tables

**Table 1:** Comparison between material properties of Cu and relevant Cu–Ni alloys [45,46].

Metal Alloy (Composition, at.%)	Melting Point (°C)	Electrical Resistivity, ρ (×10^−8^ Ωm)	Thermal Conductivity, k (Wm^−1^K^−1^)	Ratio, ρ/k (×10^−8^ Ωm^2^W^−1^ K)
Cu	1080	8.5	340	0.025
Cu–Ni (40%/60%)	1200	54.5	49.3	1.105
Cu–Ni (70%/30%)	1150	39	60.5	0.645

**Table 2:** Comparison between material properties of Al and relevant Al–Ni alloys [45,46,47,48].

Metal Alloy (Composition, at.%)	Melting Point (°C)	Electrical Resistivity, ρ (×10^−8^ Ωm)	Thermal Conductivity, k (Wm^−1^K^−1^)	Ratio, ρ/k (×10^−8^ Ωm^2^W^−1^K)
Al	650	10.5	200	0.053
Al–Ni (50%/50%)	1680	10	72	0.139
Ni_3_Al (75%/25%)	1400	73	36	2.028
